# Expanding the phenotype of *THRB*: a range of macular dystrophies as the major clinical manifestations in patients with a dominant splicing variant

**DOI:** 10.3389/fcell.2023.1197744

**Published:** 2023-07-21

**Authors:** Elena Fernández-Suárez, María González-del Pozo, Alejandro García-Núñez, Cristina Méndez-Vidal, Marta Martín-Sánchez, José Manuel Mejías-Carrasco, Manuel Ramos-Jiménez, María José Morillo-Sánchez, Enrique Rodríguez-de la Rúa, Salud Borrego, Guillermo Antiñolo

**Affiliations:** ^1^ Department of Maternofetal Medicine, Genetics and Reproduction, Institute of Biomedicine of Seville (IBiS), University Hospital Virgen del Rocío/Spanish National Research Council (CSIC)/University of Seville, Seville, Spain; ^2^ Center for Biomedical Network Research on Rare Diseases (CIBERER), Seville, Spain; ^3^ Department of Clinical Neurophysiology, University Hospital Virgen Macarena, Seville, Spain; ^4^ Department of Ophthalmology, University Hospital Virgen Macarena, Seville, Spain; ^5^ RETICS Patología Ocular, OFTARED, Instituto de Salud Carlos III, Madrid, Spain

**Keywords:** cone dystrophy, Stargardt disease, macular dystrophy, autosomal dominant, *THRB*, thyroid hormone resistance, splicing variant

## Abstract

Inherited retinal dystrophies (IRDs) are a clinically and genetically heterogeneous group of disorders that often severely impair vision. Some patients manifest poor central vision as the first symptom due to cone-dysfunction, which is consistent with cone dystrophy (COD), Stargardt disease (STGD), or macular dystrophy (MD) among others. Here, we aimed to identify the genetic cause of autosomal dominant COD in one family. WGS was performed in 3 affected and 1 unaffected individual using the TruSeq Nano DNA library kit and the NovaSeq 6,000 platform (Illumina). Data analysis identified a novel spliceogenic variant (c.283 + 1G>A) in the thyroid hormone receptor beta gene (*THRB*) as the candidate disease-associated variant. Further genetic analysis revealed the presence of the same heterozygous variant segregating in two additional unrelated dominant pedigrees including 9 affected individuals with a diagnosis of COD (1), STGD (4), MD (3) and unclear phenotype (1). *THRB* has been previously reported as a causal gene for autosomal dominant and recessive thyroid hormone resistance syndrome beta (RTHβ); however, none of the IRD patients exhibited RTHβ. Genotype-phenotype correlations showed that RTHβ can be caused by both truncating and missense variants, which are mainly located at the 3′ (C-terminal/ligand-binding) region, which is common to both *THRB* isoforms (TRβ1 and TRβ2). In contrast, the c.283 + 1G>A variant is predicted to disrupt a splice site in the 5′-region of the gene that encodes the N-terminal domain of the TRβ1 isoform protein, leaving the TRβ2 isoform intact, which would explain the phenotypic variability observed between RTHβ and IRD patients. Interestingly, although monochromacy or cone response alterations have already been described in a few RTHβ patients, herein we report the first genetic association between a pathogenic variant in *THRB* and non-syndromic IRDs. We thereby expand the phenotype of *THRB* pathogenic variants including COD, STGD, or MD as the main clinical manifestation, which also reflects the extraordinary complexity of retinal functions mediated by the different *THRB* isoforms.

## 1 Introduction

Inherited retinal dystrophies (IRDs) constitute a complex group of rare disorders with extreme clinical and genetic heterogeneity, which cause visual loss due to improper development, dysfunction, or premature death of the retinal photoreceptors or the retinal pigment epithelial cells (Schneider et al., 2022). More than 50 major subtypes of IRDs have been described so far, which can be clinically classified based on the photoreceptor type that is primarily involved in disease pathogenesis. In patients starting with rods degeneration like retinitis pigmentosa (RP), the initial clinical symptoms are night blindness and tunnel vision since these cells are primarily located along the peripheral edges of the retina and are responsible for vision at low light levels (scotopic vision) (Himawan et al., 2019). In contrast, cones are mostly concentrated in the macula, the central portion of the retina and, are active at higher light levels (photopic vision). These cells are critical for visual acuity and color discrimination (Himawan et al., 2019). This means that the affected individuals with a primary cone dysfunction manifest poor central vision as the first symptom such as in cone dystrophy (COD), Stargardt disease (STGD), or macular dystrophy (MD) among others.

IRDs can be inherited as autosomal recessive (AR), autosomal dominant (AD), or X-linked (XL) disorders. Although in most cases, the disease is limited to the eye (non-syndromic), over 80 forms of syndromic IRD have been described (Tatour and Ben-Yosef, 2020). To date, more than 290 genes have been associated with some type of IRDs, (https://web.sph.uth.edu/RetNet/, accessed March 2023), of which about 50 genes are involved in cone-dominated diseases (Cremers et al., 2018). However, the overlapping causative genes and phenotypes tremendously complicate the genetic analysis of IRDs, and a relatively large percentage of affected individuals (∼40%) remain genetically unsolved after routine analyses (Martín-Sánchez et al., 2020). In this sense, the application of a global approach like whole genome sequencing (WGS) promises to increase the diagnostic yield of IRDs through the identification of challenging variants like structural variants, deep-intronic variants, or variants in novel causative genes (Gonzalez-Del Pozo et al., 2022).

**FIGURE 1:**
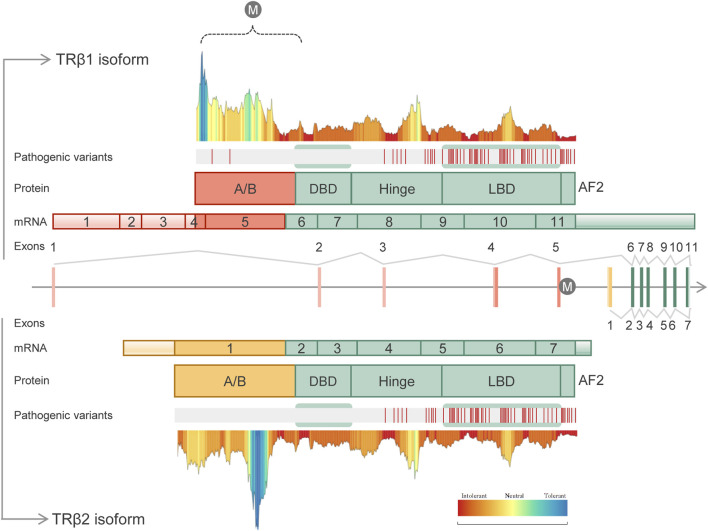
Schematic representation of the human *THRB* gene structure, transcripts, protein domains, distribution of known pathogenic variants and tolerance landscape. The identified variant (c.283 + 1G>A) is represented with the “M” letter. Two different isoforms are shown: TRβ1 (UniProtKB, P10828-1) containing 11 exons and 461 amino acids (above panel), and TRβ2 (UniProtKB, P10828-2) containing 7 exons and 476 amino acids (below panel). Both isoforms differ in the A/B domains which are encoded by specific exons. Common domains included DNA-binding domain (DBD), hinge domain, ligand-binding domain (LBD) and the AF2 domain. The distribution of the known pathogenic variants along the THRB domains was done using curated HGMD-pro data (Stenson et al., 2020). Of note, to date disease-causing variants (“DM” class) have been identified only in RTHβ individuals, being mainly located within the common LBD and hinge domains. MetaDome server (Wiel et al., 2019) (https://stuart.radboudumc.nl/metadome) was used to create the tolerance landscape of both THRB isoforms. The variant identified here in IRD patients, c.283 + 1G>A, is predicted to disrupt the A/B domain of the TRβ1 isoform (dashed bracket). In this region, only two pathogenic variants have been identified in RTHβ patients.

In the human retina, three different cones subtypes are distinguished, based on the expression of opsin proteins with different spectral sensitivities (Zhang et al., 2019). The red-opsin or L-cones are stimulated by long-wavelength light; the green-opsin or M-cones respond to medium-wavelength light; and, the blue-opsin or S-cones, are stimulated by short-wavelength light (Schmidt et al., 2019). In humans, S-cones are first specified, followed by M/L-cones. This specification is regulated by the thyroid hormones triiodothyronine (T3) and thyroxine (T4) signaling (Eldred et al., 2018), through the activation of the thyroid hormone receptor nuclear receptor (*THRB*) gene expression (Emerson et al., 2013). *THRB* gene produces two transcripts using alternative splicing: TRβ1, which is widely distributed, and TRβ2, which is limited to the cochlea, retina, and pituitary gland (Zaig et al., 2018). Both isoforms contain the typical domain structure of a nuclear receptor with an N-terminal domain (A/B) with hormone-independent transactivation activity; a central DNA-binding domain (DBD) consisting of two C4-type zinc fingers that recognized DNA motif in the regulatory regions of genes; a hinge region with a nuclear location signal; the C-terminal ligand-binding domain (LBD) with regions essential for hormone-dependent transactivation, and the AF2 domain (Tian et al., 2006) (Figure 1). These two transcripts only differ in the N-terminal domain (Zaig et al., 2018). Numerous studies in model organisms have established a role for TRβ2 during cone subtype specification (Ng et al., 2001; Roberts et al., 2005; Ng et al., 2009; Suzuki et al., 2013; Aramaki et al., 2022). In mice*,* the deletion of the specific isoform TRβ2 prompts the expression of only S-cones and the complete loss of M-cones, revealing an essential role for TRβ2 in M-cone identity (Ng et al., 2001). In addition, a wide range of studies in others model organisms, such as zebrafish or rats, also showed retinal abnormalities (McNerney and Johnston, 2021). Moreover, Eldred et al. demonstrated that in the absence of *THRB,* all cones developed into the S subtype using human retinal organoids. In addition to its role in cone opsin specification, THRB also regulates cone survival. Excess of T3 signaling through TRβ2 induces cone apoptosis (Ng et al., 2017). Taken together, these findings suggest an important role for *THRB* in cone-related disorders.

In fact, visual impairment and cones dysfunction have been noted in rare cases of thyroid hormone resistance syndrome beta (RTHβ) (Lindstedt et al., 1982; Frank-Raue et al., 2004; Weiss et al., 2012; Campi et al., 2017), a syndrome caused by variants in the *THRB* gene. RTHβ is a rare disease mainly inherited as an autosomal dominant pattern, although a few cases of autosomal recessive inheritance have been also reported (Refetoff et al., 1967; Takeda et al., 1991). RTHβ is characterized by inappropriate findings in the serum levels of free thyroid hormones (T3 and T4) with high circulating thyroid-stimulating hormone (TSH). Most RTHβ patients are considered clinically euthyroid and present a wide phenotypic variability, even among patients harboring the same variant in *THRB* (Campi et al., 2021). More than 190 truncating and missense variants have been associated with RTHβ (data from HGMD-pro, March 2023), most of which are located in the LBD (T3-binding) domain or in the contiguous hinge region (Figure 1). Interestingly, common SNPs in the intron control region (ICR) have been associated with the clinical variability in RTHβ (Alberobello et al., 2011).

Here, we identified a canonical spliceogenic variant affecting the A/B domain of the TRβ1 isoform of THRB in three unrelated Spanish families with a diagnosis of cone-dominated disorders (COD, STGD, and MD). To our knowledge, this is the first report of the identification of *THRB* variants in IRD patients, with macular degeneration as the major clinical feature, which suggests novel retinal functions of this complex gene.

## 2 Materials and methods

### 2.1 Subjects and clinical evaluation

**FIGURE 2:**
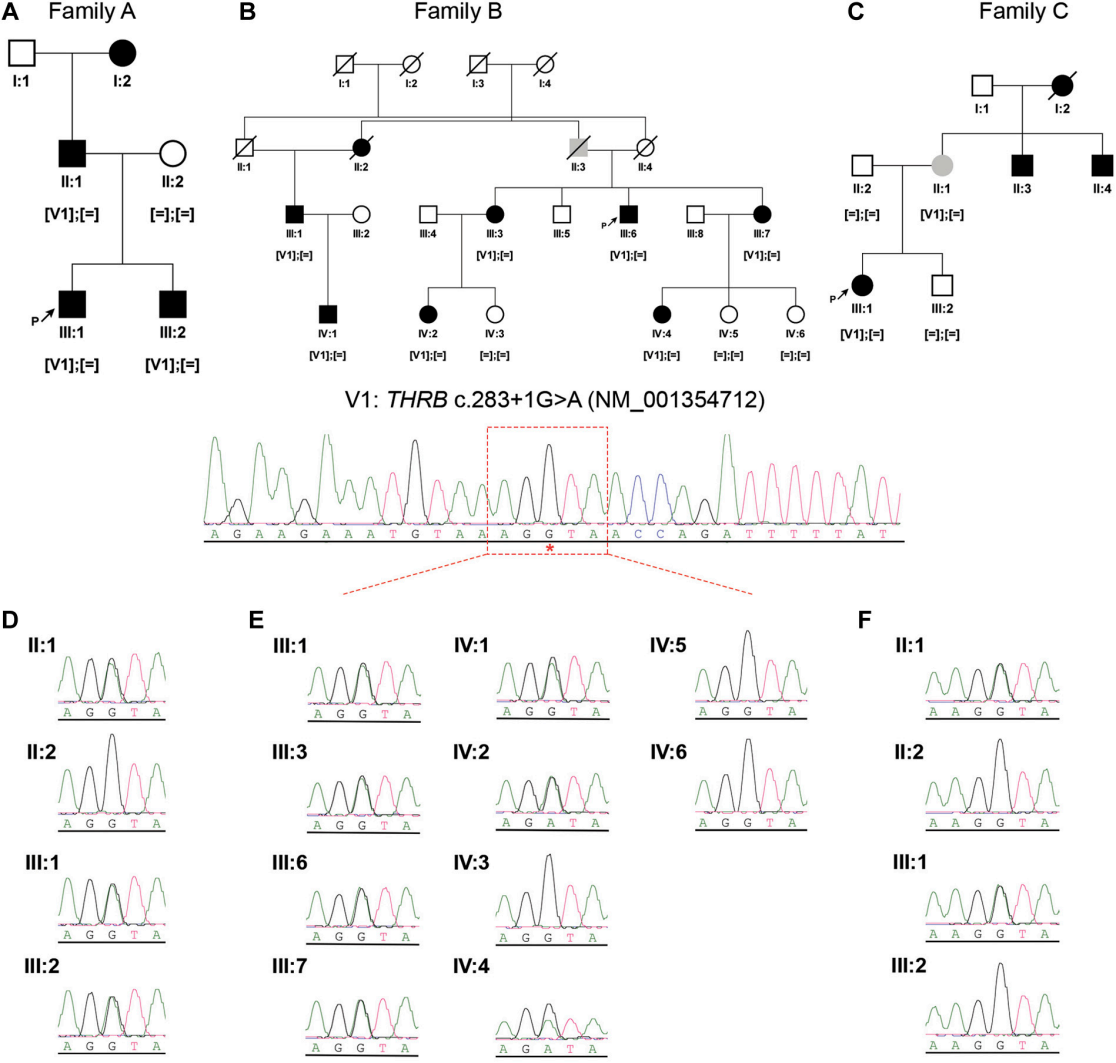
Pedigree structures and segregation analysis of the recurrent *THRB* variant (NM_001354712: c.283 + 1G>A; r.spl; NP_001341641.1: p.?) in the three IRD families **(A–C)**. Black symbols represent affected individuals, white symbols represent unaffected individuals and grey symbols represent individuals with uncertain clinical diagnosis. ‘[ = ]; [ = ]’: wild-type genotype; ‘[V1]; [ = ]’: heterozygous genotype. **(D–F)**, Sanger sequencing electropherograms and segregation results in the available members of family A **(D)**, family B **(E)** and family C **(F)**.

Three unrelated Spanish families consisting of 9 unaffected and 11 affected individuals with a presumed autosomal dominant IRD, were recruited for genetic diagnosis (Figure 2). Genomic DNA was isolated from peripheral blood using standard procedures. An informed consent form was signed by all participants or their legal guardians for clinical and genetic studies. The research was conducted in accordance with the tenets of the Declaration of Helsinki (Edinburgh, 2000), and approved by the Institutional Review Boards of the University Hospital Virgen del Rocio and the University Hospital Virgen Macarena (Seville, Spain).

All affected individuals were derived from the Ophthalmic Departments at the University Hospital Virgen Macarena and University Hospital Virgen del Rocio, and underwent a comprehensive ophthalmic examination including best corrected visual acuity (BCVA), fundus photographs, fundus fluorescein angiography (FA), optical coherence tomography (OCT), visual field, visual evoked potentials (VEP), and electroretinography (ERG).

In addition, clinical data relevant to the disease of each patient were obtained from the electronic health record (EHR), including routine blood tests showing thyroid hormones studies (free thyroxine, FT4, and thyroid-stimulating hormone, TSH).

### 2.2 Targeted NGS and mutational screening

As part of our diagnostic routine, individual III:1 from family A, individuals IV:1, III:3 and III:7 from family B and III:1 from family C, underwent targeted panel sequencing of 1,166 genes associated with different rare diseases, as previously described (Puppo Moreno et al., 2022). After a negative result using a virtual IRD panel comprising 146 associated genes (https://web.sph.uth.edu/RetNet/), the families were included in the unsolved IRD cohort.

The unsolved IRD cohort was composed of a total of 215 individuals whose genomic information was used to extract local-frequency data and to evaluate the recurrence of novel candidate variants. In fact, as the *THRB* gene was already included in the targeted diagnostic panel for RTHβ patients, we searched for the presence of likely pathogenic variants in all coding exons and its splice junctions of this gene in the unsolved IRD cohort. Prioritization of *THRB* variants was done using the prediction tools and optimized cutoffs previously described elsewhere (Gonzalez-Del Pozo et al., 2022).

### 2.3 Whole genome sequencing and genetic analysis

Individuals II:1, II:2, III:1 and III:2 from family A (Figure 2) underwent WGS by Macrogen (Seoul, Korea). DNA libraries were constructed using the TruSeq Nano DNA Library Prep Kit (Illumina, CA, United States) according to the manufacturer’s instructions. The quality of the libraries was confirmed using a 2100 Bioanalyzer (Agilent Technologies, CA, United States). WGS was performed using 2 × 150 base paired-end reads on an Illumina NovaSeq 6,000 platform. After sequencing, trimmed reads were mapped to the hg19 human reference genome using BWA-MEM (v. 0.7.17). BAM files were sorted, and duplicates were removed using Picard (v. 2.18.2). GATK (v. 4.0.5.1) was used for base quality recalibration and variant calling of single-nucleotide variants (SNVs). The variant annotation was done using SnpEff (v. 5.0e). The variant calling of structural variants (SVs) was done with Manta (v. 1.5.0) and copy number variations (CNVs) were identified by Control-FREEC (v.11.5) and PennCNV (v. 1.0.5), and both annotated using AnnotSV 2.2 online software (Geoffroy et al., 2021). The final output was a vcf file per sample and type of variants.

The WGS data prioritization was conducted using our validated pipeline as previously described (Gonzalez-Del Pozo et al., 2022). Briefly, the WGS data of the four sequenced individuals were combined using VCF combine (Vcflib). The resulting multi-sample vcf file was annotated with data from gnomaAD browser, CADD v1.6 scores, SpliceAI scores and ClinVar significance (January 2023). Variants with a minor allele frequency (MAF) less than 1% in public databases (gnomAD) were first prioritized and subsequently filtered using the customized filters. Non-splicing variants were prioritized with the prediction tools: CADD (≥22.25), MAPP (≤0.098 or absent), Grantham (≥28 or absent), and SIFT (≤0.175 or absent). The prioritization of splicing variants was done using an update of the worklflow (data unpublished) with SpliceAI (Jaganathan et al., 2019) and MaxEntScan tools (Yeo and Burge, 2004). The application of the “pedigree filtering” helped us to prioritize variants according to their zygosity and phenotype (González-Del Pozo et al., 2020). Finally, a manual curation was carried out according to the American College of Medical Genetics/Association for Molecular Pathology (ACMG/AMP) guidelines (Richards et al., 2015) using the Franklin Genoox Platform (https://franklin.genoox.com/), the clinical significance in databases such as ClinVar, LOVD, or HGMD-pro, and the number of homozygous and heterozygous (absent, 0, 1) in gnomAD, among others criteria. The nomenclature of variants was adjusted to the Human Genome Variation Society (http://varnomen.hgvs.org/) guidelines.

Candidate variants were confirmed and segregated in the available family members by PCR and direct Sanger sequencing according to the manufacturer’s protocols (3730 DNA Analyzer, Applied Biosystems, Foster City, CA, United States).

### 2.4 Protein structural analysis of TRβ1 and its predicted splicing impacts

To evaluate the effect of alternative splice process derivate from c.283 + 1G>A variant, a three-dimensional modeling for TRβ1*,* TRβ1-skipping exon 5, and TRβ1-skipping exons 5 and 6 were conducted. The modeling of TRβ1-skipping exon 5 was performed using IntFOLD (McGuffin et al., 2023) (Integrated Protein Structure and Function Prediction Server, University of Reading, https://www.reading.ac.uk/bioinf/). The three-dimensional modeling for the THRβ-skipping exons 5 y 6 were conducted using PEP-FOLD4 (Lamiable et al., 2016; Rey et al., 2023) (https://bioserv.rpbs.univ-paris-diderot.fr/services/PEP-FOLD4/) due to the small size of the resulting peptide.

### 2.5 Statistical analysis

In order to investigate if the prevalence of the variant is significantly increased in affected individuals as compared to controls, a statistical analysis was performed using RStudio 2022.02.3 and R version 4.1.3. The chi-square test, considering a significant value of *p* < 0.05, was performed to compare these categorical variables.

## 3 Results

### 3.1 Clinical features in family A

**FIGURE 3:**
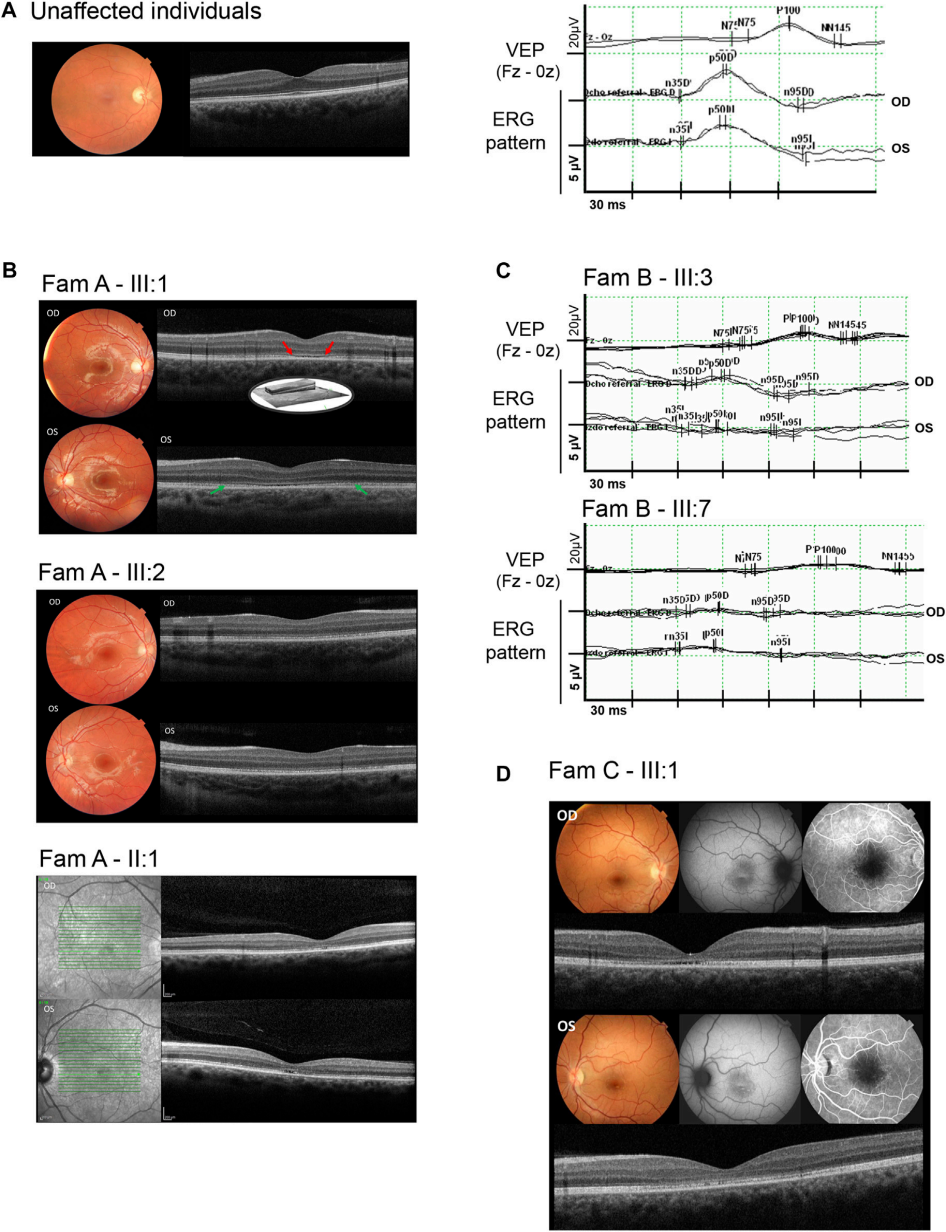
Ophthalmic characterization of some of the IRD individuals who harbored the *THRB* variant. **(A)**, Clinical characterization of an unaffected control individual including normal color fundus photographs, optical coherence tomography, visual evoked potentials (VEP), and ERG pattern responses in both eyes. **(B)**, Clinical characterization of affected individuals from family A, including color fundus photographs (individuals III:1, and III:2) showing orange-yellowish lesions in the fovea; fundus autofluorescence imaging (individual II:1), and optical coherence tomography (individuals III:1, III:2 and II:1) showing foveal cavitation (red arrows) and the disruption of the photoreceptor and retinal pigment epithelium layers (green arrows). **(C)**, Visual evoked potentials (VEP) showing prolonged P100 latency and ERG pattern with abnormal responses in both eyes (OD: right eye and OS: left eye) from individuals III:3 and III:7 of family B, **(D)**, Clinical characterization of the affected individual from family C (individual III:1) showing macular atrophy, loss of the ellipsoid zone, foveal cavitation, hyper- and hypoautofluorescent changes in fovea and disruption of the photoreceptor cells layer.

Family A was of Spanish origin. Affected individuals received a clinical diagnosis of COD and had a suspected autosomal dominant pedigree due to the existence of multiple affected individuals of both genders in three consecutive generations (Figure 2). The first symptom in all affected individuals was a decrease in visual acuity, but individuals III:1 and III:2 had a childhood-onset (6–7 years respectively) whereas in individual II:1 the symptoms began during adulthood, showing a milder phenotype. Ophthalmic examination revealed orange-yellowish lesions in the macula, foveal cavitation and the disruption of the photoreceptor and retinal pigment epithelium layers (Figure 3). ERG of these individuals were consistent with central vision defects showing pathological pattern ERG with abnormal responses in both eyes and prolonged P-100 latency in the VEP. Additional clinical findings of the sequenced patients are reported in Table 1.

**TABLE 1:** Clinical characteristics of the individuals harboring the c.283 + 1G>A variant in *THRB*.

Family and pedigree subject	Onset age/age at time of evaluation (years)	First symptom	Ophthalmic examination	ERG	Clinical diagnosis	Additional manifestations
Fam-A II:1	Adulthood	Decreased of visual acuity	Loss of central vision and mild fundus alterations	NA	COD	Milder phenotype
Fam-A III:1	7/9	Decreased of visual acuity	Loss of central vision, photopic response alteration, foveal cavitation, orange-yellowish lesion in fovea	VEP for central vision bilaterally affected (prolonged P100 latency)	COD	NA
				Pathological pERG bilaterally		
			Disruption of the photoreceptor outer segment junction	Affected cone responses		
				Impairment of photopic retinal responses		
Fam-A III:2	6/8	Decreased of visual acuity	Loss of central vision, photopic response alteration, foveal cavitation, orange-yellowish lesion in fovea	NA	COD	NA
Fam-B III:1	Childhood/39	Decreased of visual acuity	NA	NA	STGD	NA
Fam-B III:3	50/54	Decreased of visual acuity			STGD	Multinodular goiter; Hypodense nodules with peripheral rim calcification; Papillary Thyroid Carcinoma
			Loss of central vision and decreased retinal thickness at the macular level	VEP for the function of central vision altered bilaterally (prolonged P100 latency)		Total thyroidectomy
			Disruption of the photoreceptor outer segment junction, mild subfoveal retinal detachment, atrophic lesions, cataracts	pERG: altered bilaterally		Hypothyroidism; Depression; Increased body weight
				No alterations in the function of the RPE, rods or cones		Type II diabetes mellitus
						Hypertension; Hypertriglyceridemia
						Osteoarthritis
Fam-B III:6	Childhood/29	Decreased of visual acuity	Decreased visual acuity, loss of central vision, photophobia, and macular atrophy	NA	MD	Goiter; Headache
Fam-B III:7	Childhood/42	Decreased of visual acuity	Loss of central vision, retinal flecks, chorioretinal atrophy with hypertrophy of the RPE, atrophic macules with hyper- and hypoautofluorescent changes.	VEP for the function of central vision altered bilaterally in a moderate-severe degree (prolonged P100 latency)	STGD	Type II diabetes mellitus; Increased body weight
				pERG: severely altered bilaterally		
				No alterations in the function of the RPE, rods or cones		
Fam-B IV:1	11/11	Decreased of visual acuity	Blurred vision, myopia, and early macular lesions	NA	MD	Migraine
Fam-B IV:2	31/35	Decreased of visual acuity	Loss of central vision, hyperfluorescence at the fovea, RPE atrophy, bright yellow/orange distributed spots consistent with lipofuscin deposits, salt-and-pepper RPE mottling and pigment clumping, macular atrophy, and thinning of the fovea	NA	STGD	Hearing impairment (left ear); Depression; Anxiety
Fam-B IV:4	20/27	Decreased of visual acuity	Macular scotoma, diffuse posterior pole pigmentation, dull macula, and lack of foveal reflex	NA	MD	Atopic dermatitis; Anxiety
Fam-C II:1	2 nd decade of life/55	Decreased of visual acuity	NA (refused to undergo an ophthalmological evaluation but manifested visual impairment)	NA	Unclear clinical diagnosis	Hyperthyroidism for 14 years, and then subclinic hypothyroidism; Psoriasiform dermatitis; Hyperlipidemia
Fam-C III:1	24/25	Decreased of visual acuity	Loss of central vision, photophobia	Abnormal pERG.	COD	Increased body weight; Depression; Atopic dermatitis
			Macular atrophy and loss of the ellipsoid zone, foveal cavitation, hyper- and hypoautofluorescent changes in fovea			
			RPE atrophy			

Abbreviations: COD, cone dystrophy; ERG, electroretinogram; Fam, family; MD, macular dystrophy; NA, not available; pERG, pattern ERG; RPE, retinal pigment epithelium; STGD, stargardt disease; VEP, visual evoked potentials.

### 3.2 WGS data quality

The reliability of WGS data was given by the quality parameters of the generated data. Genome sequencing in the four studied individuals of family A produced an average total yield of 88.76 Gb ± 1.34 (mean ± SD) and an average coverage of 30.63x±0.58 (mean ± SD). Only 0.92% of the bases showed a coverage less than 10x. The percentage of mapped reads and duplicated reads was 99.73% and 6.52%, respectively. The total base Q ≥ 30 was 90.27%. The Q score of 30 to a base is equivalent to the probability of an incorrect base call 1 in 1,000 times. This means that the base call accuracy is 99.9%, thus, all the reads will have zero errors and ambiguities in 90.27% of the bases. All these parameters indicated that WGS rendered high-quality data.

### 3.3 Identification and assessment of candidate variants in family A

Application of WGS in family A resulted in an average of 5,052,864 SNVs/indels, 691 CNVs, and 8,972 SVs per sample, which were annotated and filtered to identify causative variants.

**FIGURE 4:**
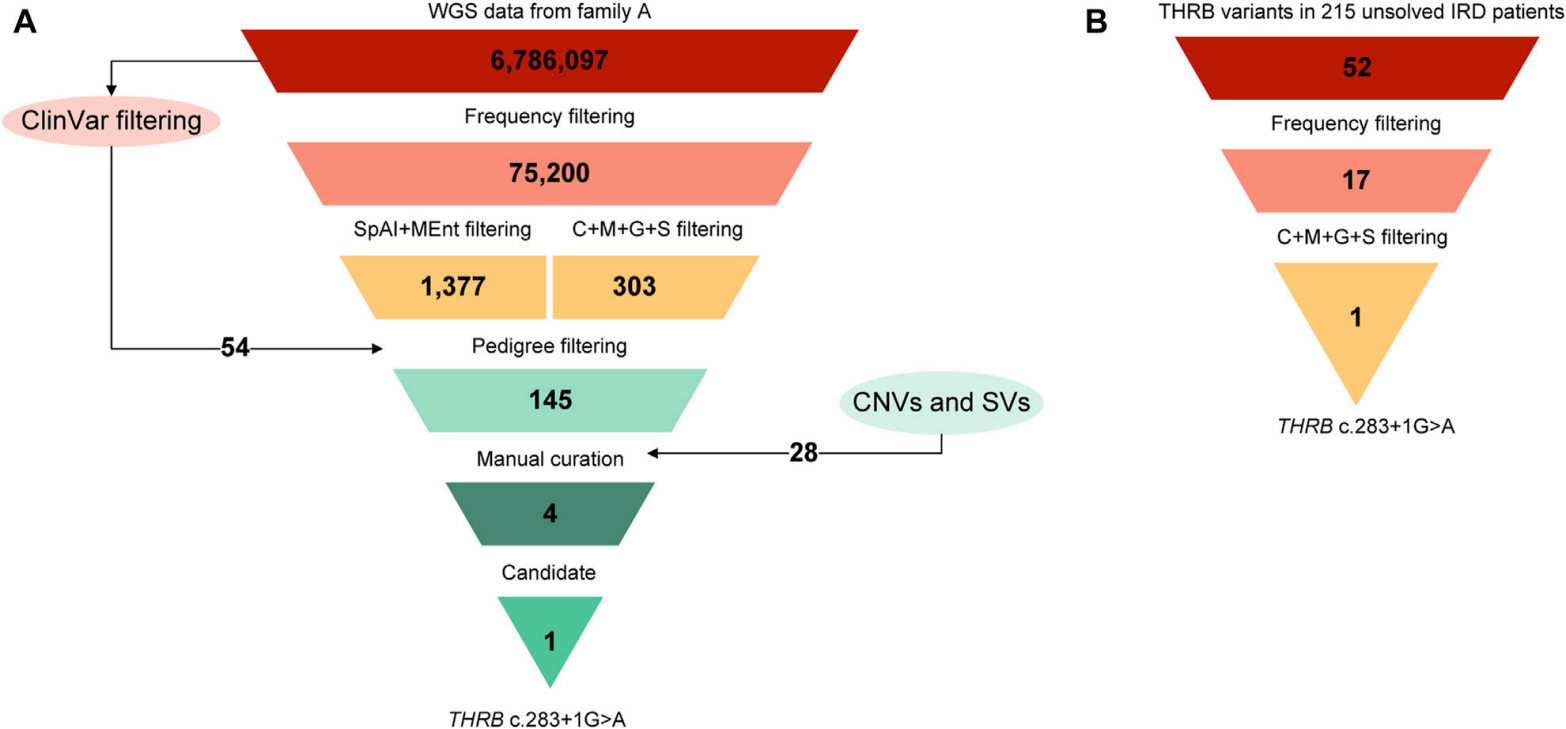
Variant filtering scheme used for the prioritization of the NGS data. **(A)** The starting point was the combined vcf file from the four sequenced individuals of family A (FamA-II:1, II:2, III:1, and III:2) that was filtered using the settings and the cutoffs described elsewhere (Gonzalez-Del Pozo et al., 2022). Briefly, variants with a MAF>0.01 in gnomAD database were filtered out except those variants classified as pathogenic (P), likely pathogenic (LP), or as conflicting interpretations of pathogenicity with P or LP entries (CIP: P/LP) in ClinVar database. Then, a combination of predictions tools including ‘CADD + MAPP + Grantham + SIFT’ (C + M + G + S) for non-splicing variants and ‘SpliceAI + MaxEntScan’ (SpAI + MEnt) for splicing variants were applied. The pedigree filtering consisted in prioritizing those variants present in the three affected individuals and absent in the unaffected individual. At this point, copy number variations (CNVs) and structural variants (SVs) were also considered. In addition, according to an autosomal dominant inheritance, variants with 2 or more heterozygous individuals in public control databases were filtered out as a first approach. Lastly, the manual curation, including ACMG/AMP classification and a comprehensive bibliography search, led to the identification of one candidate variant in this family. **(B)** Prioritization of the variant in THRB gene in the unsolved IRD cohort applying the workflow described above. The final analysis allows the identification of the THRB **(C)** 283 + 1G>A variant in families B and C.

The multi-sample vcf from family A encompassed more than six million of SNVs. After the application of the customized pipeline, we identified 75,199 rare SNVs (MAF≤0.01) and 54 SNVs with MAF>0.01 that were recovered by ClinVar filtering (Supplementary Table S1). The subsequent filters with prediction tools prioritized 1,686 variants, of which 309 variants passed “CADDv1.6 + MAPP + Grantham + SIFT” filtering and 1,377 variants were recovered by “SpliceAI + MaxEnt” filtering (Figure 4). An autosomal dominant inheritance pattern was assumed for the analysis of the family as a first approach, prioritizing those common variants in the three affected individuals and absent in the unaffected mother. After the application of this pedigree filtering 142 variants were considered for further analysis. No coding nor spliceogenic deep-intronic variants in known IRD genes were identified consistent with the disease. Additionally, after the prioritization of CNVs and SVs, only 28 variants passed the applied filters (Figure 4; Supplementary Table S1). All the filtered variants present in affected individuals were manually curated considering the ACMG/AMP classification and the number of heterozygous or homozygous in gnomAD. This curation allowed the identification of four variants (Table 2). A comprehensive bibliography search of the candidate variants allowed us to propose a novel heterozygous *THRB* variant (NM_001354712.2: c.283 + 1G>A; r.spl; NP_001341641.1: p.?) as the most likely cause of the disease due to its role in cone development in different animal models (Supplementary Table S2). Family segregation results are depicted in Figure 2.

**TABLE 2:** Variants prioritized in the family A during the application of the manual curation.

Gene	gDNA (hg19)	cDNA | protein	ACMG/AMP class	MAF (gnomAD)	OMIM phenotype	Phenotype MIM number	Inh	Role in cone function
*THRB*	chr3:g.24231564C>T	NM_001354712.2: c.283 + 1G>A | NP_001341641.1: p.?	LP	NA	RTHβ	#188570	AD	Yes
					RTHβ	#274300	AR	
					RTHβ, selective pituitary	#145650	AD	
*GRM6*	chr5:g.178416383G>A	NM_000843.4: c.1036C>T | NP_000834.2: p.Arg346*	P	NA	CSNB 1B	#257270	AR	No
*STK38*	chr6:g.36507933G>A	NM_001305102.1: c.47C>T | NP_001292031.1: p.Thr16Ile	VUS	0.000115	NA	NA	NA	No
*TDRD9*	chr14:g.104441854T>G	NM_153046.3: c.975T>G | NP_694591.2: p.Tyr325*	LP	NA	SPGF30	#618110	AR	No

Abbreviations: AD, autosomal dominant; AR, autosomal recessive; CSNB 1B, congenital stationary night blindness 1B; Inh, inheritance; LP, likely pathogenic; MAF, minor allele frequency; NA, not available; P, pathogenic; RTHβ, resistance to thyroid hormone β; SPGF30, spermatogenic failure-30; VUS, variant of unknown significance.

According to the ACMG/AMP guidelines, although the variant affects the canonical splicing +1 position, the PVS1 rule cannot be applied because this requires the existence of a prior association between loss-of-function variants and the IRD phenotype, being here reported for first time. However, the application of the PM2 rule (absent from controls), and the PP3 rule (multiple lines of computational evidence support a deleterious effect on the gene or gene product), resulted in a “VUS” classification. Moreover, all splicing prediction tools used in our pipeline predicted a disruption of the canonical donor splicing site with a high score (SpliceAI = 0.98 and MaxEnt variation = 57.74%), and the CADD tool also displayed an elevated score (CADD_phred v1.6 = 34). Based on previous studies (Anna and Monika, 2018) exon skipping is the most frequent consequence of canonical splice site mutations, therefore the identified variant is expected to produce the skipping of exon 5 or exons 5 and 6 of *THRB*, or even a mix of aberrantly spliced transcripts*.* Using *in silico* predictions, we hypothesized the skipping of exon 5, which would produce a protein with an abnormal N-terminal domain conserving intact the rest of protein. Instead, the skipping of the two exons (exons 5 and 6) would create a premature stop codon producing an incomplete protein. Three-dimensional modeling of these predicted consequences showed conformational differences in the protein folding (Supplementary Figure S1).

Moreover, in order to explain the clinical heterogeneity between the three affected individuals, additional genetic variants in this and other genes were considered. Remarkably, the individual FamA-II:1 harbored *in trans* with the c.283 + 1G>A variant, two common SNPs, rs2596622 and rs2596623, in the ICR of *THRB,* previously associated with clinical heterogeneity in RTHβ patients (Alberobello et al., 2011).

### 3.4 Mutational screening of *THRB*

Expanded genetic analysis of the *THRB* gene in the 215 unsolved patients from our IRD cohort allowed the identification of the same variant (c.283 + 1G>A) in two additional unrelated families clinically diagnosed with different autosomal dominant cone diseases (Figure 4). Sanger sequencing revealed segregation of the *THRB* variant with the disease in 8 affected, 6 unaffected and 1 individual with unclear phenotype (Figure 2). No alternative candidate variants (SNVs nor CNVs) explaining the retinal condition were identified in any case.

Remarkably, the c.283 + 1G>A variant has been identified in 11 alleles in our IRD cohort whereas it was totally absent in public control databases including gnomAD, Bravo and CSVS (Pena-Chilet et al., 2021). Therefore, the frequency of the variant in IRD patients was significantly higher than in control individuals (*p*-value = 2.2·10^−16^, Chi-squared test). The application of two additional pathogenic ACMG/AMP rules, PP1 (cosegregation with the disease in multiple affected family members) and PS4 (the prevalence of the variant in affected individuals was significantly increased compared to the prevalence in controls) led us to re-classify this variant as “likely pathogenic” (Class IV).

### 3.5 Genotype-phenotype correlation and thyroid hormones studies

Combining the phenotypic characteristics of the 12 affected individuals, we delineated the updated ophthalmologic spectrum of phenotypes associated with the splicing variant c.283 + 1G>A in *THRB*. Autosomal dominant retinal dystrophies such as COD (*n* = 4), STGD (*n* = 4) or MD (*n* = 3) represented the major clinical diagnoses that motivated the genetic testing (Table 1). One individual (FamC-II:1) who carried the variant (Figure 2), refused to be reevaluated. Affected individuals manifested a reduction in visual acuity as first symptom being the age of onset variable between 6 and 31 years old. Fundus evaluation showed photoreceptor layer thinning at macular area, and foveal cavitation consistent with central visual impairment in most of the patients (Figure 3). Regarding the neuro-ophthalmological studies, VEP revealed pathological changes consisting of prolonged P100 latency and abnormal ERG pattern responses (Figure 3). No color vision alterations were reported among the individuals of our cohort.

Routine blood tests, including thyroid-stimulating hormone (TSH), and free thyroxine (FT4) levels, were conducted in 8 out of 12 patients. Patients showed normal FT4 values (mean: 1.14 ± 0.18 ng/dL; normal range, 0.89–1.80 ng/dL), and normal TSH values (mean: 2.88 ± 2.31 µUI/mL (0.40–4.00 µUI/mL), except two who showed high TSH values (4.24 and 7.72 µUI/mL respectively). Regrettably, free triiodothyronine (FT3) was only measured in one patient, and it was normal (Supplementary Table S3). Of note, none of the affected individuals received a clinical diagnosis of RTHβ because most remain in a euthyroid state with no specific endocrine test recommendations. However, related features such as multinodular goiter (*n* = 2), increased body weight (*n* = 3), hyperlipidemia (*n* = 2), hypothyroidism (*n* = 1), type II diabetes mellitus (*n* = 2) or hearing impairment (*n* = 1) were observed. Additional clinical manifestations included skin diseases (*n* = 3), anxiety and depression (*n* = 3). More details about the clinical characterization are summarized in Table 1 and in Supplementary Table S3.

## 4 Discussion

We present one Spanish family with dominant IRD harboring a novel sequence variant in the *THRB* gene (c.283 + 1G>A). An expanded genetic analysis of the *THRB* gene in our unsolved IRD cohort, resulted in the identification of the same variant in two additional unrelated families. The unusually high frequency of the variant in our population, suggests a possible founder effect. This genetic information enabled us to propose a new genotype-phenotype correlation between cone-dominated diseases and the presence of variants in this gene.

Numerous studies have shown a role for *THRB* in cone development and survival using a wide range of model organisms (Supplementary Table S2). However, monoallelic or biallelic *THRB* variants have only been associated with generalized or selective pituitary RTHβ in humans (Refetoff et al., 1967; Gershengorn and Weintraub, 1975; Weiss et al., 1993; Adams et al., 1994; Ferrara et al., 2012; Ortiga-Carvalho et al., 2014), with just a few patients showing visual impairment (Frank-Raue et al., 2004; Weiss et al., 2012). Here, we have expanded the phenotype of *THRB* with the identification of a putatively spliceogenic variant associated with retinal degeneration, as the main clinical outcome in three unsolved IRD families. Although thyroid involvement was barely observed, multinodular goiter, and hyper- or hypothyroidism were observed in three patients. This suggests that RTHβ and IRD might not be fully independent clinical entities, but different manifestations of the same syndromic disease in which the expressivity of the different clinical features may vary from patient to patient. Additionally, some patients manifested a metabolic disorder, including increased body weight, type II diabetes mellitus, and hyperlipidemia Also, mental health conditions (anxiety and depression) and, skin diseases (atopic dermatitis and psoriasiform dermatitis) were also reported. Altogether these data suggest that *THRB* is associated with a highly variable clinical expression, even within the same family, suggesting the role of phenotype modifiers in THRB-associated conditions or variable *THRB* expression. In this sense, variations in *THRB* enhancer sequences that influence the expression of TRβ2 in both retina and pituitary have been proposed to explain tissue-specific phenotypes (Jones et al., 2007; Alberobello et al., 2011; Liu et al., 2023). Individual II:1 from family A harbored two SNPs within a putative regulatory intronic region that, although still controversial (Zaig et al., 2018), have been hypothesized to modify the expression of THRβ isoforms (Alberobello et al., 2011). Remarkably, individual II:1 presented a milder phenotype than his sons (III:1 and III:2), which could associate with the presence of these SNPs; however, further studies are needed to corroborate this hypothesis.

Despite the phenotypic variability observed in IRD patients harboring the c.283 + 1G>A variant, all individuals were characterized by primary cone dysfunction. This is shown as a reduction in the thickness of the photoreceptor layer, and the foveal cavitation that is characteristically present in most of the patients of our cohort. The visual outcome for these 11 patients was, on average, worse in comparison to RTHβ patients harboring dominant-negative variants in *THRB* (Campi et al., 2017), as none of those patients (*n* = 27) met the clinical criteria to be diagnosed of MD, STGD or COD, and only color vision changes and a 10% reduction of the cone-response to a single flash of light during the photopic ERG were observed. It was intriguing that previously described dominant variants in *THRB* caused variable but closely related ocular symptoms compared with the recessive variants which consisted mainly of differences in the cone-subtype specification (Weiss et al., 2012). Our patients did not manifest color vision alterations, and regarding the neuro-ophthalmological studies, VEP changes in the form of prolonged P100 latency, and the abnormal pattern electroretinogram responses were consistent with severe central visual defects showing a clinical diagnosis varied from COD (*n* = 4), STGD (*n* = 4), and MD (*n* = 3). One carrier refused to conduct an ophthalmological revaluation but manifested visual impairment since the second decade of life according to her EHR.

Genotype-phenotype correlations showed that RTHβ can be caused by both truncating and missense variants, which are mainly located at the common domains in the TRβ1 and TRβ2 isoforms, the LBD domain and the contiguous hinge region (Weiss et al., 1993). In contrast, the variant identified here, c.283 + 1G>A, is predicted to disrupt the N-terminal A/B domain of the TRβ1 isoform. This region shows promoter- and cell-specific activity (Aranda and Pascual, 2001) and is target for numerous post-translational modifications including phosphorylation, SUMOylation, and acetylation (Weikum et al., 2018). We hypothesized that the c.283 + 1G>A variant could impact retinal-specific functions mainly involved in cone viability without a major affectation of cone subtype specification that is mediated by TRβ2. In fact, a recent study experimentally demonstrates that TRβ1-knockout mice displayed only minor changes in opsin photopigment expression (Ng et al., 2023), resembling what we observed in our patients. Authors suggest that TRβ1, the predominant TRβ isoform at mature ages, may have a role in the survival of both cone photoreceptors and retinal pigment epithelium cells (Ng et al., 2023), as described in other retinal degeneration models (Ma et al., 2014; Ma et al., 2017; Ma et al., 2022), and now in the IRD patients of our cohort. Of note, only two putatively pathogenic variants have been identified in the TRβ1 N-terminal domain in patients with congenital hypothyroidism and thyroid dysgenesis (Zhou et al., 2018). However, the young age of these patients, the lack of an ophthalmologic evaluation, and the VUS status of these variants hampered the proper establishment of genotype-phenotype correlations. Here, we report the first likely pathogenic splicing variant in *THRB*, which also affects only the TRβ1 isoform, reflecting both the extraordinary intolerance to variation of this gene and the high complexity of retinal functions mediated by the different *THRB* isoforms. Hence, it will be of interest to screen this and other *THRB* variants in IRD patients from different populations in order to evaluate the burden of *THRB* variants in additional IRD cohorts worldwide.

Also, dominant negative effects produced by *THRB* variants in RTHβ patients have been described and are possibly explained by the formation of heterodimers between normal and functionally inactive mutant receptor, which would diminish the activity of the resultant thyroid receptor (TR). This would reduce the amount of normal and potentially functional TR-T3 complexes and, therefore, higher concentrations of the hormone would be required to produce sufficient amount of hormone-saturated TR homodimers (Takeda et al., 1991). In fact, in this scenario, higher demands of thyroid hormones could trigger the activation of compensatory mechanisms like the increase of T3 levels, leading to cone apoptosis if this occurs in certain tissues like the retina (Ma et al., 2014; Ng et al., 2017). This is in line with previous findings that associated high T3 levels with an increased risk of age-related macular degeneration in human populations (Chaker et al., 2015; Gopinath et al., 2016). This is supported by the ocular phenotype described in our patients, who presented the photoreceptor layer thinning and the foveal cavitation without color vision alterations.

On the other hand, our work has also highlighted the importance of reanalysis of NGS data before proceeding with the generation of new genetic data using sequencing protocols such as WES or WGS in unsolved patients. In fact, the *THRB* gene was already included in the targeted diagnostic approach for these patients, but it was not routinely analyzed in IRD patients due to the lack of association. We conclude that sequencing of a smaller set of genomic regions during the diagnostic routine, may show some discovery potential for the identification of genes not yet associated to a particular disease, but routinely sequenced during the diagnostic process, resulting in the establishment of novel genotype-phenotype correlations.

To sum up, given the reported eye findings in a group of nearly 30 RTHβ patients (Weiss et al., 2012; Campi et al., 2017) and the demonstration that ablation of *THRB* in animal models induces retinal changes mainly consisting of cone differentiation defects, ophthalmological monitoring should be recommended together with an endocrine evaluation in patients with suspected THRB-associated syndrome. Similarly, likely pathogenic variants in the *THRB* gene, especially in the TRβ1 specific exons, should also be considered as disease-causing in patients with clinically diagnosed macular dystrophies, cone-dystrophy, or Stargardt disease with or without extra-ocular manifestations. We thereby expanded the phenotype of *THRB* pathogenic variants including a spectrum of IRD as the main clinical manifestation.

## Figures and Tables

**Table:** 

**ACMG/AMP**	American College Of Medical Genetics/Association For Molecular Pathology
**AD**	Autosomal Dominant
**AR**	Autosomal Recessive
**BCVA**	Best Corrected Visual Acuity
**BWA**	Burrows-Wheeler Alignment Tool
**C + M + G + S**	Cadd + Mapp + Grantham + Sift
**CIP:P/LP**	Conflicting Interpretations Of Pathogenicity With P Or LP Entries
**CNVs**	Copy Number Variations
**COD**	Cone Dystrophy
**CSNB1B**	Congenital Stationary Night Blindness 1B
**CSVS**	Collaborative Spanish Variant Server
**DBD**	DNA-Biding Domain
**EHR**	Electronic Health Record
**ERG**	Electroretinography
**FA**	Fundus Fluorescein Angiography
**fam**	Family
**FT3**	Free Triiodothyronine
**FT4**	Free Thyroxine
**GATK**	Genome Analysis Toolkit
**gDNA**	Genomic DNA
**ICR**	Intron Control Region
**Inh**	Inheritance
**IRD**	Inherited Retinal Dystrophies
**LBD**	Ligand-Binding Domain
**LP**	Likely Pathogenic
**MAF**	Minor Allele Frequency
**MD**	Macular Dystrophy
**NA**	Not Available
**NGS**	Next-generation sequencing
**OCT**	Optical Coherence Tomography
**P**	Pathogenic
**pERG**	Pattern Electroretinography
**PM2**	Pathogenic Moderate Criteria 2
**PP1**	Pathogenic Supporting Criteria 1
**PP3**	Pathogenic Supporting Criteria 3
**PS4**	Pathogenic Strong Criteria
**PVS1**	Pathogenic Very Strong Criteria 1
**RP**	Retinitis Pigmentosa
**RPE**	Retinal Pigment Epithelium
**RTHβ**	Thyroid Hormone Resistance Syndrome Beta
**SNP**	Single Nucleotide Polymorphism
**SNVs**	Single-Nucleotide Variants
**SpAI + MEnt**	SpliceAI + MaxEntScan
**SPGF30**	Spermatogenic Failure-30
**STGD**	Stargardt Disease
**SVs**	Structural Variants
**T3**	Triiodothyronine
**T4**	Thyroxine
**THRB**	Thyroid Hormone Receptor Beta Gene
**TR**	Thyroid Receptor
**TRβ1**	Thyroid Hormone Receptor Beta Isoform 1 Protein
**TRβ2**	Thyroid Hormone Receptor Beta Isoform 2 Protein
**TSH**	Thyroid-Stimulating Hormone
**VCF**	Variant Call Format
**VEP**	Visual Evoked Potentials
**VUS**	Variant Of Uncertain Significance
**WGS**	Whole Genome Sequencing
**XL**	X-Linked

## Data Availability

The datasets for this article are not publicly available due to concerns regarding participant/patient anonymity. Requests to access the datasets should be directed to the corresponding authors. The prioritized variant was submitted to ClinVar database under the accession ID: SCV003845203.
