# Supplementary material for: Expanding the phenotype of THRB: a range of macular dystrophies as the major clinical manifestations in patients with a dominant splicing variant
Source: Front Cell Dev Biol. 2023 Jul 21;11:1197744. doi: 10.3389/fcell.2023.1197744 (PMC10401274; doi:10.3389/fcell.2023.1197744)
Supplement: Supplementary file 1 [file Table2.PDF]

Expanding the phenotype of *THRB*: a range of macular dystrophies as the major clinical manifestations in patients with a dominant splicing variant
Fernández-Suárez E et al., Front. Cell Dev. Biol. 2023

Supplementary Table 2 Different *THRB in vivo* and *in vitro* knock-out and knock-down models and its phenotypic effect.

MODEL ORGANISM	REFERENCE NAME METHOD	*THRB* EFFECT	OBSERVED PHENOTYPE	REFERENCE
***Rattus norvegicus* (Wistar)**	HG rats<br>Chemical antithyroid solution	T3/T4-deficient	Decreased retinal growth during the perinatal stage.	(Sevilla-Romero et al., 2002)
***Rattus norvegicus* (Long-Evans)**	Treatment with propylthiouracil	T3/T4-deficient	Reduced amplitude of green-flicker ERGs: impaired function of M-cone photoreceptors	(Boyes et al., 2018)
Mus musculus	Thrb$^{b2cre/b2cre}$	Deletion of TRβ2	Poorly distinguished cones that tend to homogeneity and as expected all express *Opn1sw* but lack *Opn1mw*	(Aramaki et al., 2022)
	Thrb$^{tm2Df/tm2Df}$	Deletion of TRβ2	Significant loss of functional M-cones.	(Ng et al., 2001)
	TRβ$^{WT/\Delta337T}$<br>TRβ $^{\Delta337T/\Delta337T}$<br>+ 0.1% methimazole	TR unable to bind T3	The 337T mutation increased S-opsin expression	(Pessoa et al., 2008)
	TRβ$^{WT/E457A}$<br>TRβ$^{E457A/E457A}$	Blocking binding of coactivators to the AF2 domain	Decrease (30%) M-opsins in both the dorsal and ventral retina	
	TRβ$^{PV/PV}$	T3 cannot bind to TRβ	All cones expressed S-opsin	(Roberts et al., 2006)
	Thrb^{b1}	K.O. Thrb^{b1} lacZ mice that express galactosidase instead of TRβ1	Minor changes in opsin photopigment expression and normal photopic ERG responses	(Ng et al., 2023)
Danio rerio	6BP+1 mutant<br>Frameshift mutation in exon 1 of TRβ2 by CRISPR/Cas9	Deletion of TRβ2	Anticipated LWS-cone loss	(Deveau et al., 2020)
	3BP mutant<br>Frame single codon deletion in exon 1 of TRβ2 by CRISPR/Cas9	Deletes Tyr61, leaves the DNA binding site and ligand binding site intact	Cone peaks shifted significantly to shorter wavelengths	
	Morpholino<br>trβ2 knockdown	Decrease of trβ2 expression	Reduced number of L cones and increase in UV cones	(Suzuki et al., 2013)
	Morpholino<br>Thrβ knockout in *tbx2b*$^{lor/lor}$ background	Deletion of Thrβ in Tbx2 Hypomorphic allele	Overabundance of rods and a small number of UV cones	(DuVal and Allison, 2018)

	Morpholino Thrβ knockdown in *tbx2b*$^{fby/fby}$ background	Deletion of Thrβ in Tbx2 Null allele	Have lots of rods and a near-complete absence of UV cones	
***Homo sapiens* (organoids)**	Thrβ null mutant Knockout by CRISPR/Cas9	Completely ablate THRB function	Complete conversion of all cones to the S type	(Eldred et al., 2018)

Supplementary Table 1 references

Aramaki, M., Wu, X., Liu, H., Liu, Y., Cho, Y.W., Song, M., et al. (2022). Transcriptional control of cone photoreceptor diversity by a thyroid hormone receptor. *Proc Natl Acad Sci U S A* 119(49)**,** e2209884119. doi: 10.1073/pnas.2209884119.

Boyes, W.K., Degn, L., George, B.J., and Gilbert, M.E. (2018). Moderate perinatal thyroid hormone insufficiency alters visual system function in adult rats. *Neurotoxicology* 67**,** 73-83. doi: 10.1016/j.neuro.2018.04.013.

Deveau, C., Jiao, X., Suzuki, S.C., Krishnakumar, A., Yoshimatsu, T., Hejtmancik, J.F., et al. (2020). Thyroid hormone receptor beta mutations alter photoreceptor development and function in Danio rerio (zebrafish). *PLoS Genet* 16(6)**,** e1008869. doi: 10.1371/journal.pgen.1008869.

DuVal, M.G., and Allison, W.T. (2018). Photoreceptor Progenitors Depend Upon Coordination of gdf6a, thrbeta, and tbx2b to Generate Precise Populations of Cone Photoreceptor Subtypes. *Invest Ophthalmol Vis Sci* 59(15)**,** 6089-6101. doi: 10.1167/iovs.18-24461.

Eldred, K.C., Hadyniak, S.E., Hussey, K.A., Brenerman, B., Zhang, P.W., Chamling, X., et al. (2018). Thyroid hormone signaling specifies cone subtypes in human retinal organoids. *Science* 362(6411). doi: 10.1126/science.aau6348.

Ng, L., Hurley, J.B., Dierks, B., Srinivas, M., Salto, C., Vennstrom, B., et al. (2001). A thyroid hormone receptor that is required for the development of green cone photoreceptors. *Nat Genet* 27(1)**,** 94-98. doi: 10.1038/83829.

Ng, L., Liu, H., Liu, Y., and Forrest, D. (2023). Biphasic expression of thyroid hormone receptor TRbeta1 in mammalian retina and anterior ocular tissues. *Front Endocrinol (Lausanne)* 14**,** 1174600. doi: 10.3389/fendo.2023.1174600.

Pessoa, C.N., Santiago, L.A., Santiago, D.A., Machado, D.S., Rocha, F.A., Ventura, D.F., et al. (2008). Thyroid hormone action is required for normal cone opsin expression during mouse retinal development. *Invest Ophthalmol Vis Sci* 49(5)**,** 2039-2045. doi: 10.1167/iovs.07-0908.

Roberts, M.R., Srinivas, M., Forrest, D., Morreale de Escobar, G., and Reh, T.A. (2006). Making the gradient: thyroid hormone regulates cone opsin expression in the developing mouse retina. *Proc Natl Acad Sci U S A* 103(16)**,** 6218-6223. doi: 10.1073/pnas.0509981103.

Sevilla-Romero, E., Munoz, A., and Pinazo-Duran, M.D. (2002). Low thyroid hormone levels impair the perinatal development of the rat retina. *Ophthalmic Res* 34(4)**,** 181-191. doi: 10.1159/000063885.

Expanding the phenotype of *THRB*: a range of macular dystrophies as the major clinical manifestations in patients with a dominant splicing variant
Fernández-Suárez E et al., Front. Cell Dev. Biol. 2023

Suzuki, S.C., Bleckert, A., Williams, P.R., Takechi, M., Kawamura, S., and Wong, R.O. (2013). Cone photoreceptor types in zebrafish are generated by symmetric terminal divisions of dedicated precursors. *Proc Natl Acad Sci U S A* 110(37), 15109-15114. doi: 10.1073/pnas.1303551110.
