# Supplementary material for: Expanding the phenotype of THRB: a range of macular dystrophies as the major clinical manifestations in patients with a dominant splicing variant
Source: Front Cell Dev Biol. 2023 Jul 21;11:1197744. doi: 10.3389/fcell.2023.1197744 (PMC10401274; doi:10.3389/fcell.2023.1197744)
Supplement: Supplementary file 2 [file Table3.pdf]

Expanding the phenotype of *THRB*: a range of macular dystrophies as the major clinical manifestations in patients with a dominant splicing variant
Fernández-Suárez E et al., Front. Cell Dev. Biol. 2023

Supplementary Table 3 Clinical and biochemical parameters of available patients harboring the c.283+1G>A variant in THRB gene. Abnormal values are in bold numbers.

FAMILY AND PEDIGREE SUBJECT	CLINICAL DIAGNOSIS	EXTAOCULAR MANIFESTATIONS	FT4 ng/dl (0.89 - 1.80)	TSH µUI/ml (0.40 - 4.00)
**Fam-B III:3**	STGD	Multinodular goiter; Hypodense nodules with peripheral rim calcification; Papillary Thyroid Carcinoma; Total thyroidectomy; Hypothyroidism; Depression; Increased body weight; Type II diabetes mellitus; Hypertension; Hypertriglyceridemia; Osteoarthritis.	1.35†	0.92†
**Fam-B III:6**	MD	Goiter; Headache.	1.03	1.22
**Fam-B III:7**	STGD	Type II diabetes mellitus; Increased body weight.	1.15	3.39
**Fam-B IV:1**	MD	Migraine.	1.36	**4.24**
**Fam-B IV:2**	STGD	Hearing impairment (left ear); Depression; Anxiety.	1.06	0.85
**Fam-B IV:4**	MD	Atopic dermatitis; Anxiety.	N.A.	1.9
**Fam-C II:1**	Unclear clinical diagnosis	Hyperthyroidism for 14 years, and then subclinic hypothyroidism; Psoriasiform dermatitis; Hyperlipidemia.	0.91<br><br>FT3: 1ng/ml (1.6 – 55)	**7.72**
**Fam-C III:1**	COD	Increased body weight; Depression; Atopic dermatitis.	N.A.	2.76
**Mean ± standard deviation levels**			1.14 ± 0.18	2.88 ± 2.31

Abbreviations: COD, Cone dystrophy; FT3, free triiodothyronine; FT4, free thyroxine; MD, macular dystrophy; N.A., not available data; STGD, Stargardt disease; TSH, thyroid stimulating hormone. †Values obtained before the thyroidectomy.
