# Supplementary material for: Expanding the phenotype of THRB: a range of macular dystrophies as the major clinical manifestations in patients with a dominant splicing variant
Source: Front Cell Dev Biol. 2023 Jul 21;11:1197744. doi: 10.3389/fcell.2023.1197744 (PMC10401274; doi:10.3389/fcell.2023.1197744)
Supplement: Supplementary file 4 [file Image1.PDF]

Expanding the phenotype of *THRB*: a range of macular dystrophies as the major clinical manifestations in patients with a dominant splicing variant
Fernández-Suárez E et al., Front. Cell Dev. Biol. 2023

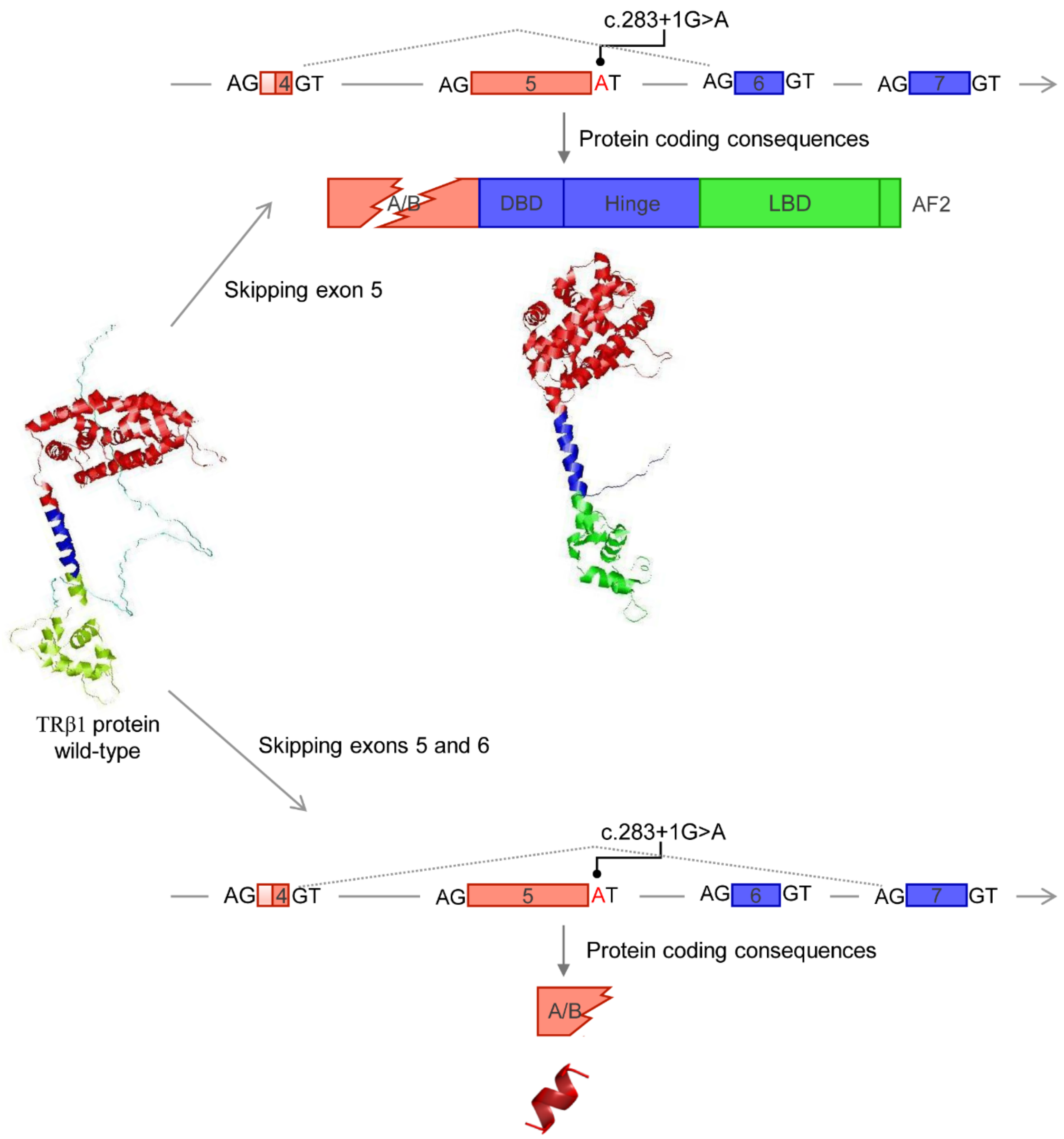

Supplementary figure S1. Schematic representation of the hypothetical consequences of the *THRB* variant. *In silico* prediction of the effect of the spliceogenic variant identified in TRβ1 isoform (NM_001354712.2: c.283+1G>A) showed two scenarios. **(A),** The skipping of exon 5 disrupts the A/B domain, leaving a protein of 374 amino acids with a secondary structure different from the wild-type protein. The DNA-binding domain (DBD), hinge domain and ligand-binding domain (LBD) are not affected. **(B),** The skipping of exons 5 and 6 results in a premature codon stop that produces only a small peptide. The 3D models of the proteins were generated using the IntFOLD and PEP-FOLD4 online servers.
